# Therapeutic potential of bixin on inflammation: a mini review

**DOI:** 10.3389/fnut.2023.1209248

**Published:** 2023-09-13

**Authors:** Saminathan Shadisvaaran, Kok-Yong Chin, Shahida Mohd-Said, Xin-Fang Leong

**Affiliations:** ^1^Department of Craniofacial Diagnostics and Biosciences, Faculty of Dentistry, Universiti Kebangsaan Malaysia, Kuala Lumpur, Malaysia; ^2^Department of Pharmacology, Faculty of Medicine, Universiti Kebangsaan Malaysia, Cheras, Malaysia; ^3^Department of Restorative Dentistry, Faculty of Dentistry, Universiti Kebangsaan Malaysia, Kuala Lumpur, Malaysia

**Keywords:** annatto, *Bixa orellana*, bixin, inflammation, safety

## Abstract

Chronic inflammation is the underlying mechanism for many diseases. Thus, inflammatory signaling pathways are valuable targets for new treatment modalities. Natural products have gained interest as a potential source of bioactive compounds which provide health benefits in combating inflammatory-related diseases. Recent reports have linked the medicinal values of *Bixa orellana* L. with its anti-inflammatory activities. Therefore, this review aims to examine the therapeutic potential of bixin, a major bioactive constituent found in the seeds of *B. orellana*, on inflammatory-related diseases based on existing *in vitro* and *in vivo* evidence. Additionally, the anti-inflammatory mechanism of bixin via signaling pathways is explored and possible toxic effects are addressed. The findings suggest that bixin may ameliorate inflammation via inhibition of toll-like receptor 4/nuclear factor-kappa B (TLR4/NF-κB), phosphoinositide 3-kinase/protein kinase B (PI3K/Akt) and thioredoxin-interacting protein/NOD-like receptor protein 3 (TXNIP/NLRP3) inflammasome mechanisms. More well-planned clinical studies should be performed to verify its effectiveness and safety profile.

## 1. Introduction

Inflammation is an essential component of the body’s defense mechanism to protect tissues from acute injuries as well as chronic diseases. It aims to remove the causative agents and damaged tissues for tissue healing and repair. The causes of inflammation may include pathogens (e.g., bacteria and viruses), physical agents (e.g., burns, radiation), chemicals (e.g., drugs, toxins) and malfunctioning immunological reactions (e.g., rheumatoid arthritis). Various cell types of the host immune system are involved (neutrophils, lymphocytes, macrophages) during inflammation with the release of inflammatory mediators such as histamine, bradykinin, eicosanoids, cytokines, and growth factors (1).

If the harmful causative agent is not to be eradicated, or there is a disturbance in the healing process, acute inflammation may evolve into chronic inflammation. Often, this may lead to the pathogenesis of inflammatory-related diseases such as cardiovascular diseases, diabetes mellitus, metabolic syndrome, cancer, respiratory diseases, and musculoskeletal diseases (2). Anti-inflammatory drugs such as steroids and non-steroidal agents have been widely used to treat these diseases. However, the administration of these medications has been linked to various adverse effects and they are expensive. There has been a growing demand in using medicinal plants and their biologically active components for treating and preventing diseases due to their affordable cost, safety, and efficacy.

*Bixa orellana* L. belongs to the Bixaceae family, also known as achiote, is a shrub native to tropical American countries (3, 4). Its cultivation has expanded to the Caribbean, African, and Asian regions. The traditional use of *B. orellana* in alleviating inflammation was reported. Infusion and decoction of *B. orellana* leaves have been used to treat sore throat, fever, bronchitis, conjunctivitis, gastric ulcer, and rheumatism. The fruit pup is used on burn-injured skin to prevent formation of blisters and sores. The seeds have been used for treating bronchitis and healing wounds in addition to be antibiotic and expectorant (5, 6).

The pigment extracted from the seeds is commercially known as annatto. Annatto is used as seasoning and as coloring to impart yellow, orange-red color to various food products. Various methods are used to improve the yield and stability of annatto (7, 8). The seeds are regarded to be the most valuable plant part contributing to promising health benefits. Phyotchemicals isolated from the seeds are of unique importance. Bixin and norbixin are the major coloured pigments isolated from the pericarp of *B. orellana* seeds, in which bixin accounts for 80% of the total carotenoids (9). Bixin and norbixin are apocarotenoids. In seed, oxidative cleavage of lycopene produces bixin aldehyde, which is then dehydrogenased to form norbixin. Bixin is synthesized from norbixin in the presence of methyltrasnferase enzyme (10). Carotenoids such as beta carotene, cryptoxanthin, lutein, zeaxanthin, methyl bixin have been identified in the seed (11). Farnesylacetone, geranylgeranyl octadecenoate and geranylgeranyl formate are examples of terpenoids found in the seed (11). Tocotrienols are terpenoid chromanols. *B.orellana* seed is a rich source of tocotrienols, in which δ-tocotrienol predominates (12).

It was estimated that 14,500 tons of *B. orellana* seeds are produced globally. Two thirds of the annual production is marketed as dried seeds while the remaining as colorant (11). Previous studies have documented that the seeds of *B. orellana* possess various pharmacological properties, including antioxidant (13), antimicrobial (14), bone protective (15), nephroprotective (16), hypoglycemic (17), hypocholesterolemia (18), anticancer (19), and anti-inflammatory (18). While the other bioactive compounds from the seeds like tocotrienol have been reviewed extensively, bixin, which is unique to this plant receives less attention for its medicinal benefits. Furthermore, the protective effect of bixin against inflammation via modulation of toll-like receptor 4/nuclear factor-kappa B (TLR4/NF-κB), phosphoinositide 3-kinase/protein kinase B (PI3K/Akt) and thioredoxin-interacting protein/NOD-like receptor protein 3 (TXNIP/NLRP3) inflammasome is yet to be reviewed. Therefore, in this mini review, we focused on the anti-inflammatory actions of bixin via regulation of signaling pathways in inflammation-related diseases, and to highlight its potential toxicity.

## 2. Literature search

A literature search was performed in the Scopus and PubMed databases. The following keywords were searched: “*Bixa orellana* OR annatto OR bixin” AND “inflammation.” The full texts of relevant original research articles written in English were retrieved after screening the titles and abstracts. No time limitation was considered in the present review.

## 3. Anti-inflammatory effects of bixin on inflammation-related diseases

### 3.1. Skin inflammation

Antinociceptive and anti-inflammatory effect of bixin (15, 30 mg/kg, oral) was determined using carrageenan-induced paw edema rat model (20). The pain relief ability of bixin was observed at 30 mg/kg through the delayed response latency to thermal stimulus and reduced number of formalin-induced flinches. Furthermore, pretreatment of bixin prior to carrageenan injection was reported to reduce the migration of neutrophils as indicated by a reduction of myeloperoxidase activity in rat’s paw. Tao et al. (21) investigated the protective effect of bixin (200 mg/kg, intraperitoneal) on the skin against solar ultraviolet (UV)-induced acute photodamage in hairless mice. Injection of bixin 48 h before UV exposure (4.4 J/cm^2^ UVA + 240 mJ/cm^2^ UVB radiation, 24 h) downregulated the expression of inflammatory markers at the protein and mRNA levels. In addition, bixin could attenuate the epidermal thickness and oxidative DNA damage.

### 3.2. Cardiovascular inflammation

A clinical trial assessed the protective effect of bixin (1.2 mg/kg) or norbixin (0.06 mg/kg) added to a high-fat high-cholesterol diet on postprandial biochemical, oxidative stress and inflammatory markers in 12 healthy subjects. Each participant received three test meals (placebo, bixin, or norbixin) at three different turns with an interval of at least 2 days. The results revealed a significant reduction of serum lipid oxidation and pro-inflammatory cytokines such as interleukin (IL)-1, IL-6 and tumor necrosis factor-alpha (TNF-α) in participants ingesting norbixin test meal (22). Furthermore, norbixin was shown to increase the postprandial activity of the antioxidant enzyme, glutathione peroxidase. However, the cholesterol levels were not affected by norbixin. On the other hand, bixin was only able to partially prevent lipid oxidation without much improvement in other tested parameters.

In rabbits, oral administration of bixin (10, 30, mg/kg, daily, 60 days) protected against the atherosclerotic lesion induced by a hypercholesterolemic diet (23). The beneficial effect was associated with a reduction of oxidative stress and inflammatory responses, as well as improvement of lipid profile. In another experiment, it was documented that bixin (50, 100, 200 mg/kg, daily, 14 weeks) was effective in ameliorating cardiac fibrosis, diminishing insulin resistance, and suppressing dyslipidemia in mice fed with a high-fat diet (24). The observed effects may be mediated through inhibiting the activation of NF-κB signaling pathway and attenuating the elevated serum pro-inflammatory cytokines. A protective property of bixin (40, 80 μM, 24 h) was detected in human cardiac muscle cell, H9C2, against lipopolysaccharide (LPS)-induced cardiac fibrosis (24). Bixin alleviated the over-expression of IL-1β, IL-18 and TNF-α in the presence of LPS.

### 3.3. Lung inflammation

The protective effect of bixin-loaded polymeric nanoparticles (6, 12, 18%, daily, 5 days, oral) was examined in a mouse model of cigarette smoke-induced acute lung inflammation (25). Bixin prevented the increased lymphocyte and macrophage numbers as well as TNF-α levels in bronchoalveolar lavage fluid, especially with a higher dose. Also, histopathological findings of bixin show a near-normal structure of lung parenchyma without noticeable inflammation in mice exposed to cigarette smoke. An investigation was aimed to assess the anti-inflammatory potential of bixin in allergy-induced and glucocorticoid-resistant asthmatic mice (26). The results revealed that bixin (50, 100 mg/kg, 6 or 14 days, i.p) has a potent anti-inflammatory effect in both acute and chronic asthma. Bixin significantly alleviated airway hyperresponsiveness, reversed airway remodeling, suppressed the elevated pro-inflammatory cytokines and restored steroid sensitivity.

In a study, the beneficial effects of bixin were evaluated on lung inflammation and fibrosis in mice with silica-induced lung injury (27). Bixin (200 mg/kg, every 3 days, i.p) could effectively ameliorate the histological abnormalities of the lung tissue with a reduction in inflammatory cell infiltration and the size of fibrotic nodules. Besides, bixin had subdued the phosphorylation of p-65 subunit and overexpression of pro-inflammatory cytokines. Similarly, intraperitoneal injection of bixin (200 mg/kg) diminished the NF-κB inflammatory response and oxidative damage in mechanical ventilation-evoked lung injury (28). Treatment with bixin before ventilation reversed the histological alterations in the lung, suggesting a moderating role of bixin in pulmonary inflammation.

### 3.4. Renal inflammation

Supplementation of bixin (100, 200 mg/kg, 4 weeks, intragastrical) reduced the serum concentration of creatinine, urea, and uric acid in the carbon tetrachloride (CCl_4_)-treated mice. Bixin restored renal function in mice by attenuating histological abnormalities, collagen deposition and fibrosis in the kidney. Increased expression of protein levels of inflammatory cytokines and nucleus translocation of NF-κB induced by CCl_4_ was suppressed by bixin (29).

### 3.5. Hepatic inflammation

Bixin (200 mg/kg, intraperitoneal) significantly reduced serum lipid levels and liver enzymes, alanine aminotransferase and aspartate aminotransferase in mice fed with a high-fat diet for 25 weeks. Moreover, bixin was shown to diminish the infiltration of inflammatory cells and lipid droplets under microscopic examination. Bixin also suppressed the upregulated expression of inflammatory cytokines as well as inhibiting the activation of NF-κB in high-fat diet-induced liver steatosis in mice (30).

### 3.6. Eye inflammation

Bis-retinoid N-retinyl-N-retinylidene ethanolamine (A2E) is a by-product of visual cycle. Accumulation of A2E in retinal pigment epithelium (RPE) has been implicated in age-related macular degeneration with retinal inflammation and angiogenesis (31). It was seen that treating porcine RPE cells with norbixin (20 μM) decreased A2E-stimulated NF-κB activation (32). Additionally, norbixin modulated the expression of IL-6 and IL-8 by inhibiting their up-regulation following the induction by A2E. In the same study, RPE cells were used to assess the effect of norbixin on the angiogenic factors. Matrix metallopeptidases (MMPs) are regulators of inflammation (33). Vascular endothelial growth factor (VEGF) has effect on vascular permeability and neoangiogenesis (34). It was observed that norbixin effectively influenced the angiogenesis, by enhancing and decreasing the mRNA expression of MMP9 and VEGF, respectively (32).

### 3.7. Neuroinflammation

Multiple sclerosis (MS) is a chronic autoimmune and neurodegenerative disease. The pathological features of MS comprise demyelination, gliosis, axonal loss and inflammation (35). Experimental autoimmune encephalomyelitis (EAE) is the most used experimental model for human MS (35). Yu et al. (36) evaluated the anti-inflammatory effect of bixin in mice with EAE. It was demonstrated that EAE markedly augmented inflammatory cells infiltration and inflammation score as well as microglia activation in the brain. These pathological changes in EAE mice were improved with the administration of bixin (100 mg/kg/day for 18 days). Moreover, bixin reduced spinal cord demyelination and axon degeneration in EAE mice. The role of bixin in neuroinflammation may be identified through a decreased serum level of pro-inflammatory cytokines. The results were further supported by the molecular studies, in which bixin suppressed the overexpression of pro-inflammatory cytokines and promoted the up-regulation of the anti-inflammatory cytokine, IL-10. Table 1 summarizes the effects of bixin on inflammation using various disease models.

**Table 1:** Summary of animal studies on bixin with anti-inflammatory effect.

Author (reference)	Treatment and control	Experimental model	Treatment dosage and administration	Findings
Pacheco et al. (20)	**Treatment**: bixin **Negative control**: corn oil (vehicle) **Positive control**: dexamethasone (1 mg/kg)	Carrageenan-induced paw edema in male Wistar rats	15, 30 mg/kg, 1 h before carrageenan injection, oral	↓ MPO activity in rat paw at 30 mg/kg vs. negative control
Tao et al. (21)	**Treatment**: bixin **Negative control**: corn oil (vehicle) **Positive control**: no	Solar UV-induced skin damage in SKH-1 mice	200 mg/kg, 48 h before UV exposure, i.p.	↓ IL-6, TNF-α, MMP-9 levels in skin tissue vs. negative control
Somacal et al. (23)	**Treatment**: bixin **Negative control**: no treatment **Positive control**: simvastatin (15 mg/kg)	Cholesterol diet-induced atherogenesis in New Zealand white rabbits	10, 30, 100 mg/kg/day for 60 days, oral	↓ serum TNF-α levels at 10 or 30 mg/kg vs. negative control ↓ serum IL-6 levels at 10 mg/kg vs. negative control
Xu and Kong (24)	**Treatment**: bixin **Negative control**: no treatment **Positive control**: no	High-fat diet (HFD)-induced cardiac injury in male C57BL/6 mice	50, 100, 200 mg/kg/day for 14 weeks, oral	↓ serum IL-1β, IL-18 and TNF-α levels vs. negative control
Figueiredo-Junior et al. (25)	**Treatment**: bixin-loaded nanoparticles **Negative control**: no treatment **Positive control**: no	Cigarette smoke-induced acute lung inflammation in male C57BL/6 mice	100 μL of 6, 12, 18% daily for 5 days, oral	↓ TNF-α, MPO levels in BALF at 12 or 18% vs. negative control ↓ MMP-9 activity in lung tissue at 18% vs. negative control
Zhu et al. (26)	**Treatment**: bixin **Negative control**: no treatment **Positive control**: no	Ovalbumin (OVA)-induced acute asthma in female Balb/c mice	50, 100 mg/kg/day for 6 days, i.p.	↓ IL-1β, IL-5, IL-6, IL-13 levels in BALF vs. negative control
Zhu et al. (26)	**Treatment**: bixin **Negative control**: no treatment **Positive control**: no	OVA/CFA-induced dexamethasone-resistant asthma in female Balb/c mice	100 mg/kg/day for 6 days, i.p.	↓ IL-6, IL-17, TNF-α and IFN-γ levels in BALF vs. negative control
Zhu et al. (26)	**Treatment**: bixin **Negative control**: no treatment **Positive control**: no	OVA-induced chronic asthma in female Balb/c mice	50, 100 mg/kg/day for 14 days, i.p.	↓ IL-5 and IL-13 levels in BALF vs. negative control
Xue et al. (27)	**Treatment:** bixin **Negative control:** no treatment **Positive control:** no	Silica-induced chronic lung injury in male C57BL/6 mice	200 mg/kg, every 3 days (starting from 72 h before silica instillation) until the end of the experiment (day 7, day 28, day 56), i.p.	↓ IL-6, TNF-α, p-P65 levels in lung tissue vs. negative control
Tao et al. (28)	**Treatment:** bixin **Negative control:** no treatment **Positive control:** no	Mechanical ventilation-induced acute lung injury in SKH-1 mice	200 mg/kg, 72 h before ventilation, i.p.	↓ IL-6, TNF-α in BALF and ↓ p-P65 level in lung tissue vs. negative control
Ma et al. (29)	**Treatment**: bixin **Negative control**: no treatment **Positive control**: no	Carbon tetrachloride-induced renal fibrosis in male ICR mice	100, 200 mg/kg/day for 4 weeks, intragastric	↓ IL-1β and TNF-α levels in kidney tissue vs. negative control
Tao et al. (30)	**Treatment**: bixin **Negative control**: no treatment **Positive control**: no	HFD-induced liver steatosis in Nrf2 wild-type and knockout mice	200 mg/kg, every 3 days from the 12th week after HFD feeding for 25 weeks, i.p.	↓ IL-6, TNF-α, p-P65 levels in liver tissue vs. negative control
Yu et al. (36)	**Treatment**: bixin **Negative control**: no treatment **Positive control**: no	Myelin oligodendrocyte glycoprotein_35–55_ peptide-induced experimental autoimmune encephalomyelitis in female C57BL/6 mice	100 mg/kg/day for 18 days, intragastric	↓ IL-1β, IL-17, IL-18, IFN-γ in serum and spinal cord tissue vs. negative control ↓ TNF-α, IL-6, and IL-8, and ↑ IL-10 in spinal cord vs. negative control

BALF, bronchoalveolar lavage fluid; CFA, complete Freund’s adjuvant; IFN-γ, interferon-gamma; IL, interleukin; i.p., intraperitoneal; MMP-9, metalloproteinases-9; MPO, myeloperoxidase; Nrf2, nuclear factor-erythroid factor 2-related factor 2;TNF-α, tumor necrosis factor-alpha; UV, ultraviolet; vs., versus.

## 4. Anti-inflammatory mechanism of bixin via modulation of signaling pathways

**Figure 1:**
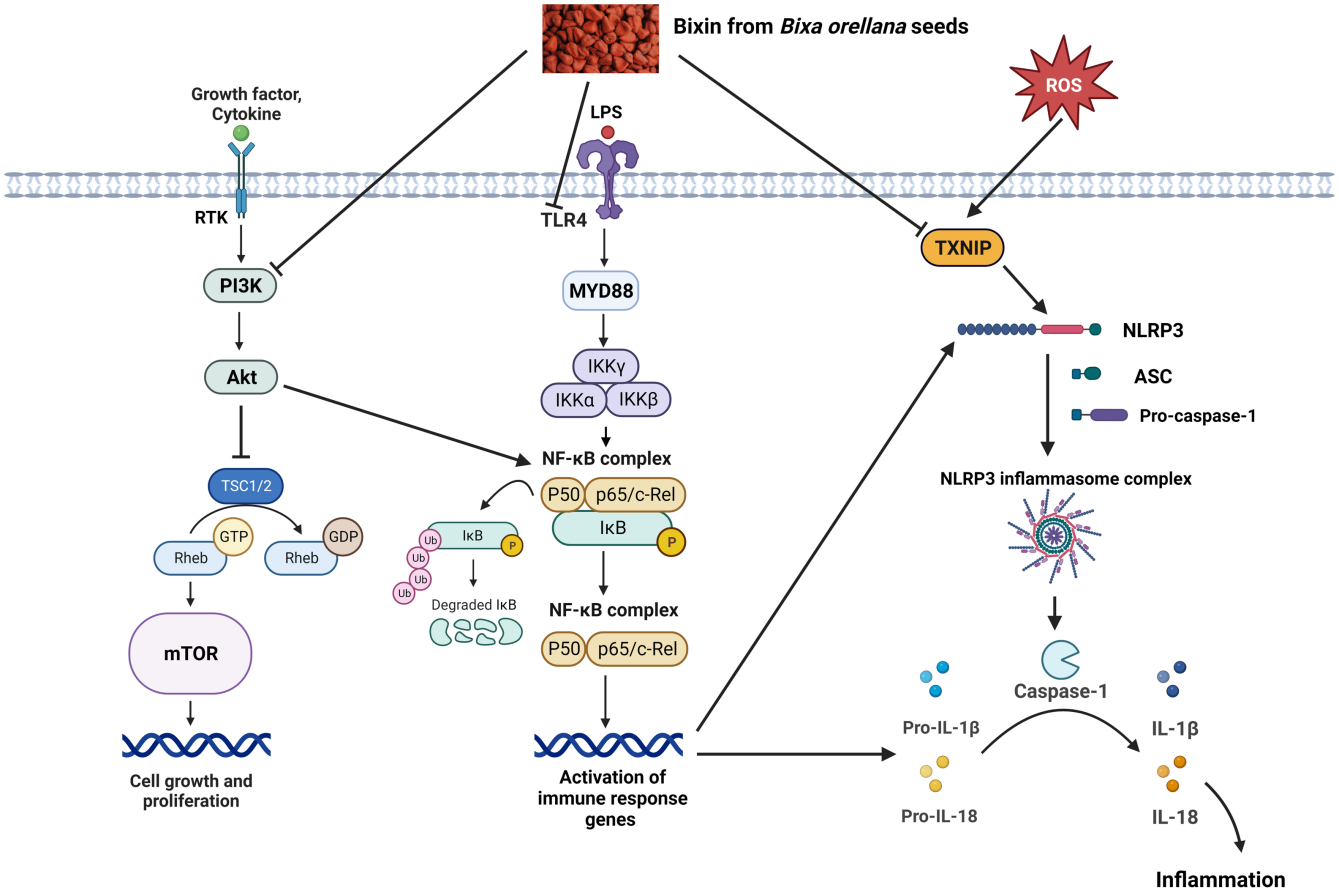
Proposed protective effects of bixin on inflammation via modulation of PI3K/Akt, TLR4/NF-κB and TXNIP/NLRP3 inflammasome signaling pathways. ASC, apoptosis-associated speck-like protein containing a caspase recruitment domain; Akt, protein kinase B; IκB, inhibitor of nuclear factor kappa B; IκK, IκB kinase; IL, interleukin; LPS, lipopolysaccharides; mTOR, mammalian target of rapamycin; MYD88, myeloid differentiation factor 88; NF-κB, nuclear factor kappa B; NLRP3, NOD-like receptor protein 3; PI3k, phosphoinositide 3-kinase; Rheb, Ras homolog enriched in brain; ROS, reactive oxygen species; RTK, receptor tyrosine kinase; TLR4, toll-like receptor 4; TSC, tuberous sclerosis complex; TXNIP, thioredoxin-interacting protein, ⊥, suppress.

Studies involving signaling pathways have reported that inflammation mediated by cytokines is an important aspect in the development of inflammatory-related conditions. As a result, regulation of the inflammatory response is an essential element in the prevention and/or treatment of diseases (37). The anti-inflammatory mechanism of bixin, via modulation of TLR4/NF-κB, PI3K/Akt and TXNIP/NLRP3 inflammasome pathways are discussed and depicted in Figure 1.

### 4.1. TLR4/NF-κB pathway

Toll-like receptors (TLRs) are commonly known as pattern recognition receptors for their ability to detect the presence of pathogens and induce immune responses (38). TLRs transmit signals via the myeloid differentiation factor 88 (MyD88)-dependent pathway, or the MyD88-independent pathway. All TLRs mediate the downstream signaling pathway via MyD88, excluding TLR3 (39). Activation of TLR4 may stimulate NF-κB, resulting in the production of pro-inflammatory cytokines. NF-κB activation requires the phosphorylation and degradation of inhibitory κB (IκB) protein, which is triggered by two kinases, IκB kinase α (IKKα) and IKKβ (40). It has been recognized that the NF-κB pathway plays an important role in the TLR4-mediated immunomodulatory effect.

Ma et al. (29) examined the protective effect of bixin on CCl_4_-induced renal damage. It was reported that bixin (100, 200 mg/kg/day for 4 weeks) attenuated the expression of TLR4 and MyD88 in the CCl_4_-treated mice. In addition, the activity of NF-κB p65, TNF-α and IL-1β were decreased in the kidney of mice. The results obtained indicate that bixin may ameliorate CCl_4_-induced kidney inflammation by inhibiting the TLR4/MyD88/NF-κB pathway.

Administration of bixin (40, 80 μM, 24 h) reduced inflammatory response by suppressing the protein levels and mRNA expression of pro-inflammatory cytokines in the human cardiac muscle cell line, H9C2, pre-exposed to LPS (24). Besides, it was shown that bixin treatment was able to reduce the protein expression of TLR4, MyD88, p-IKKα, p-IκBα and p-NF-κB. Similar results were observed when Xu and Kong (24) explored the cardioprotective effect of bixin on a high-fat-diet-induced cardiac injury model in C57BL/6 mice. The findings proposed that bixin has an inhibitory effect on TLR4/MyD88/NF-κB pathway, leading to a reduction of inflammatory responses.

### 4.2. PI3K/Akt pathway

PI3K/Akt pathway is a signaling pathway that enhances cell growth, proliferation, metabolism, and survival (41). These cellular functions are mediated through a multi-step process in which activation of PI3K causes translocation and phosphorylation of protein kinase B or also known as Akt (42). This pathway has many effects on downstream substrates, including regulating the inflammatory response to injury and infection.

Zhu et al. (26) reported that treatment with bixin (50, 100 mg/kg/day for 6 or 14 days) attenuated the expression of p-PI3K, p-Akt and p-mammalian target of rapamycin (mTOR) in the lungs of acute, chronic as well as steroid-resistant asthma in Balb/c mice. In the same study, the effect of bixin (10, 40, or 80 mM) on transforming growth factor (TGF)-β1-activated mouse airway epithelial MLE12 cells was investigated. It was revealed that bixin could suppress the upregulation of p-PI3K, p-Akt and p-mTOR *in vitro*. PI3K/Akt plays a contributory role in the recruitment of inflammatory cells into the respiratory airways of asthmatics. Administration of bixin was found to reduce the levels of pro-inflammatory cytokines in asthmatic mice (26). The inhibitory effect exerted by bixin on PI3K/Akt pathway suggests a potential therapeutic strategy in treating asthma and other related inflammatory diseases.

### 4.3. TXNIP/NLRP3 inflammasome pathway

TXNIP regulates the activation of NLRP3 inflammasome (43). NLRP3 inflammasome is a multiprotein complex comprising NLRP3, an apoptosis-associated speck-like protein containing a caspase recruitment domain (ASC) and effector protein precursor pro-caspase-1 (44). The TXNIP/NLRP3 pathway is linked to many inflammatory diseases. Activation of NLRP3 inflammasome leads to the generation of the active caspase-1, which stimulates the release of IL-1β and IL-18 (45).

Treatment with bixin (100 mg/kg/day for 18 days) decreased the levels of pro-inflammatory cytokines and increased the mRNA expression of anti-inflammatory cytokines in mice with EAE (36). It was documented that bixin increased mRNA expression of nuclear factor erythroid 2-related factor 2 (Nrf2) and its downstream genes in mice with encephalomyelitis. Furthermore, bixin caused a down-regulation of protein and gene expression of TXNIP, NLRP3, ASC, caspase-1, IL-1β, and IL-18. The anti-inflammatory effects evoked by bixin may occur at least in part through the modulation of the TXNIP/NLRP3 inflammasome pathway.

## 5. Potential toxicity of annatto

### 5.1. Subacute toxicity

In a study, male Swiss albino mice were given annatto extract (56, 351 mg/kg) or norbixin (0.8, 7.6, 66, 274 mg/kg) at various doses, respectively in 21 days via drinking water (46). A reduction of plasma globulin level was recorded in groups ingesting annatto and norbixin (7.6, 66, 274 mg/kg). Norbixin (66 mg/kg) reduced the plasma urea and creatinine levels compared to the control mice. The hypoglycemic effects of annatto extract (351 mg/kg) or norbixin (0.8, 7.6, 66 mg/kg) with a concomitant reduction in insulin levels were reported. In the same study, female Wistar rats given annatto extract (0.8, 7.5, 68 mg/kg) or norbixin (0.8, 8.5, 74 mg/kg) demonstrated that annatto extract (7.5 mg/kg) reduced total cholesterol level in comparison to control rats. The hyperglycemic effect was significant in female rats fed with annatto extract (7.5, 68 mg/kg) and norbixin (74 mg/kg). Increased plasma insulin level was observed in rats ingesting norbixin (46). The opposite effects were noted between rats and mice. The discrepancy may be attributed to the difference in species and sex.

In another study, an oral dose of 2,000 mg/kg annatto containing 27% bixin was administered to male and female Wistar rats for 20 days (47). All animals survived throughout the study duration. No severe abnormalities were detected in hematological, biochemical, and macroscopic investigations. Histological examination revealed that 20% of the female rats had localized apoptosis in the kidneys without a known reason. The data suggested that 540 mg/kg bixin did not induce toxicity in rats during the 28-day observation period.

### 5.2. Subchronic toxicity

Male and female Sprague–Dawley rats were given a diet incorporated with annatto at a dietary level of 0.1, 0.3 or 0.9% for 13 weeks (48). The results showed that annatto did not affect body weight, food and water intake, or ophthalmological, and hematological parameters. However, rats administered with annatto containing 91.6% norbixin at the dietary level of 0.3 or 0.9% exhibited an increased level of alkaline phosphatase, phospholipid, and total protein in addition to increased liver weight and hypertrophy. The no-observed-adverse-effect-level (NOAEL) of annatto was at 0.1% dietary level. This value approximates the average annatto intake of 69 mg/kg/day and 76 mg/kg/day for male and female rats, respectively, by oral route.

### 5.3. Teratogenicity

Female Wistar rats were administered 31.2, 62.5, 125, 250 or 500 mg/kg annatto containing 28% bixin on days 6 to 15 of pregnancy via oral gavage (49). No sign of toxicity or mortality was observed. In addition, no physical, visceral, or skeletal pathologies were reported in the offspring. Therefore, annatto may be regarded as safe in view that it has no undesirable effect on maternal health and embryo development in rats. The NOAEL of annatto was 500 mg/kg/day or equivalent to 140 mg/kg/day bixin for oral administration.

### 5.4. Genotoxicity and mutagenicity

Fernandes et al. (46) reported that consumed annatto extract (56, 351 mg/kg) or norbixin (0.8, 7.6, 66, 274 mg/kg) in 21 days via drinking water did not cause DNA breakage in the kidney and liver isolated from male Swiss albino mice.

A diet incorporated with annatto containing 30% bixin at various concentrations was given to male Swiss albino mice to examine its mutagenic and antimutagenic properties (50). The mice were fed with annatto (1,330, 5,330, 10, 670 ppm) for 7 days, and they received either normal saline (i.p., 0.9%) or cyclophosphamide (i.p., 50 mg/kg) on day 7 prior to sacrifice after 24 h. It was shown that the highest concentration of annatto caused an increase in the number of micronucleated polychromatic erythrocytes in the bone marrow of mice administered with cyclophosphamide. The results suggest that annatto does not inhibit mutagenicity but may increase the risk of mutagenic effects when used in higher concentrations.

### 5.5. Carcinogenicity

The effects of diet incorporated with annatto at different dietary levels (0.03, 0.1, 0.3%) on male F344 rats pretreated with N-nitrosodiethylamine (DEN, i.p., 200 mg/kg), a hepatic toxicant of carcinogenic, were investigated (51). The animals appeared normal and healthy throughout the study but intake of annatto containing 87.3% norbixin for 6 weeks caused an increase in liver weights for rats administered with 0.1% or 0.3% annatto. Despite that, the highest administered dosage did not promote liver tumor formation in DEN-pre-treated rats. Thus, annatto does not promote carcinogenesis.

## 6. Conclusion and future perspectives

*B. orellana* has traditionally been used to heal or prevent various diseases and to promote general well-being. Research into the therapeutic potential and mechanisms of action, especially its active constituent, bixin, has gained more attention from scientists in recent years. Some of the available preclinal studies did not have a positive control in their design (Table 1). Thus, the anti-inflammatory effects of bixin compared to standard anti-inflammatory agents cannot be appreciated. Future studies should incorporate positive controls to reaffirm the effectiveness of bixin as an anti-inflammatory agent. Past studies suggest the possible role of TLR4/NF-κB, PI3K/Akt and TXNIP/NLRP3 in moderating the anti-inflammatory effects of bixin. Nevertheless, its direct involvement in these signaling pathways needs exploration. Further investigation is warranted to determine the related pathways and to discover the molecular targets that mediate the protective effects of bixin on health.

Studies exploring the anti-inflammatory properties of bixin in humans are limited. The required bixin dosage to achieve optimal anti-inflammatory benefits in humans remains unknown and requires further investigation. Moreover, the protective effects of bixin have yet to be verified in clinical trials, and more safety assessments are needed to determine the potential adverse effects of bixin for prolonged use in humans. Therefore, more research is required to confirm their clinical efficacy and safety profile.

## Author contributions

SS performed the literature search and drafted the manuscript. X-FL performed the literature search, drafted, and revised the manuscript. K-YC and SM-S provided critical review for the manuscript. All authors contributed to the article and approved the submitted version.

## Conflict of interest

The authors declare that the research was conducted in the absence of any commercial or financial relationships that could be construed as a potential conflict of interest.

## Publisher’s note

All claims expressed in this article are solely those of the authors and do not necessarily represent those of their affiliated organizations, or those of the publisher, the editors and the reviewers. Any product that may be evaluated in this article, or claim that may be made by its manufacturer, is not guaranteed or endorsed by the publisher.
